# Identification and Function of Leucine-Rich Repeat Flightless-I-Interacting Protein 2 (LRRFIP2) in *Litopenaeus vannamei*

**DOI:** 10.1371/journal.pone.0057456

**Published:** 2013-02-28

**Authors:** Shuang Zhang, Hui Yan, Chao-Zheng Li, Yi-Hong Chen, Feng-hua Yuan, Yong-gui Chen, Shao-Ping Weng, Jian-Guo He

**Affiliations:** 1 Ministry of Education Key Laboratory of Aquatic Product Safety/State Key Laboratory of Biocontrol, School of Life Sciences, Sun Yat-sen University, Guangzhou, People’s Republic of China; 2 School of Marine Sciences, Sun Yat-sen University, Guangzhou, People’s Republic of China; Swedish University of Agricultural Sciences, Sweden

## Abstract

Leucine-rich repeat flightless-I-interacting protein 2 (LRRFIP2) is a myeloid differentiation factor 88-interacting protein with a positive regulatory function in toll-like receptor signaling. In this study, seven LRRFIP2 protein variants (LvLRRFIP2A-G) were identified in *Litopenaeus vannamei.* All the seven LvLRRFIP2 protein variants encode proteins with a DUF2051 domain. LvLRRFIP2s were upregulated in hemocytes after challenged with lipopolysaccharide, poly I:C, CpG-ODN2006, *Vibrio parahaemolyticus*, *Staphylococcus aureus*, and white spot syndrome virus (WSSV). Dual-luciferase reporter assays in *Drosophila* Schneider 2 cells revealed that LvLRRFIP2 activates the promoters of *Drosophila* and shrimp AMP genes. The knockdown of LvLRRFIP2 by RNA interference resulted in higher cumulative mortality of *L. vannamei* upon *V. parahaemolyticus* but not *S. aureus* and WSSV infections. The expression of *L. vannamei* AMP genes were reduced by dsLvLRRFIP2 interference. These results indicate that LvLRRFIP2 has an important function in antibacterials via the regulation of AMP gene expression.

## Introduction

Toll-like receptors (TLRs) have a key function in the innate immune response [1]–[3]. Myeloid differentiation factor 88 (MyD88) is the common intracellular adaptor protein located immediately downstream of most TLRs [4]. Upon lipopolysaccharide (LPS) stimulation, MyD88 can recruit specific intracellular proteins along the MyD88-IRAK-TRAF6-IκB-NFκB signal relay to regulate the activity of downstream transcription factors such as NF-κB [5]–[7].

Leucine-rich repeat flightless-I-interacting protein 2 (LRRFIP2) is a poorly characterized protein implicated in TLR responses as a MyD88-interacting protein in vertebrates [8]. Together with LRRFIP1, LRRFIP2 was first reported as a novel protein that interacts with the LRR domain of human flightless I homolog (Fliih), a negative mediator of NF-κB activity functioning by interfering MyD88-TLR4 interaction in an exposure time-dependent manner [9], [10]. In humans, LRRFIP1 and LRRFIP2 are related genes from gene duplication that can activate β-catenin-dependent transcription activity [11], [12]. LRRFIP1, which also goes by the names GC-binding factor 2 (GCF2), flightless I LRR-associated protein 1 (FLAP1), and TAR RNA-interacting protein [9], [11], [13], functions in the regulation of tumor necrosis factor-α production and type I interferon [13]–[15]. LRRFIP2 exhibits 41% sequence homology with murine FLAP1 [2]. Similar to LRRFIP1, LRRFIP2 has a positive regulatory function in TLR signaling by disrupting the interaction of MyD88 and Fliih upon LPS-induced signal transduction [8]. Most of the current knowledge about LRRFIP2 stems from human or murine studies. Therefore, this study aims to gain additional insight into the function of LRRFIP2 in invertebrates. According to the information in the NCBI database, several expressed variants of LRRFIP2 have been predicted in vertebrates and invertebrates. In crustaceans, LRRFIP2 has been found in expressed sequence tags (ESTs) and full-length cDNA database of *Caligus clemensi*, *Caligus rogercresseyi*, and *Lepeophtheirus salmonis*.

*Litopenaeus vannamei*, a crustacean species, is one of the most important economic penaeid shrimps worldwide [16]. Since the 1990s, numerous shrimp-farming countries around the world have suffered substantial economic losses because of bacterial and viral diseases [17], [18]. Therefore, studying the mechanism of *L. vannamei* immunity is necessary to design better strategies for disease prevention and control. The research conducted in our laboratory proposed that a TLR/MyD88/Tube/Pelle/TRAF6/NF-κB cascade exists in *L. vannamei* [19]–[24]. In the current study, the homolog of human LRRFIP2 was identified in *L. vannamei* and its signal transduction function was studied, especially in antibacterial response. This study is the first to report on the function of LRRFIP2 in invertebrates, which is helpful to obtain more information about the LRRFIP2 gene. Moreover, study of *L. vannamei* LRRFIP2 may be beneficial to better understand the innate immune pathway in shrimp, which would be helpful in preventing various diseases in shrimp culture.

## Materials and Methods

### Microorganisms

Gram-negative *Vibrio parahaemolyticus* were cultured in a thiosulfate-citrate-bile salts-sucrose (TCBS) agar culture medium at 30°C for 18 h. Gram-positive *Staphyloccocus. aureus* were cultured in a nutrient broth agar at 37°C for 24 h. The *V. parahaemolyticus* and *S. aureus* cells were centrifuged at 5000 g for 10 min at 4°C, washed with 1×PBS (8 g NaCl, 0.2 g KCl, 1.44 g Na_2_HPO_4_, and 0.24 g K_2_HPO_4_, diluted with dH_2_O to 1 litre and with the pH adjusted to 7.3), and then resuspended in 1xPBS. The bacterial concentation was quantified as the microbial colony-forming units per milliliter (CFU/ml) and the bacterial solution adjusted to 10^6^ CFU/ml.

The white spot syndrome virus (WSSV)-infected *L. vannamei* were collected from the Hengxing shrimp farm in Zhanjiang, Guangdong Province, China, and stored at −80°C. Muscle samples (0.1 g) from the WSSV -infected *L. vannamei* were homogenized in 1 ml of 1×PBS and centrifuged at 5000 g for 15 min at 4°C. The supernatant was filtered through a 0.45 µm membrane, and used as the WSSV inocula. TaqMan real-time PCR was used to quantify the WSSV dose as previously described [25].

### Cloning of LvLRRFIP2 cDNA

Based on three EST sequences of *L. vannamei* (GenBank accession Numbers. FE100722, FE045230, and FE139225) homologous to *Homo sapiens* LRRFIP2, primers were designed to obtain the full-length cDNA of LRRFIP2 by 5′ and 3′ rapid amplification cDNA ends (RACE) polymerase chain reaction (PCR). The cDNA template for RACE-PCR was prepared using the BD SMART RACE cDNA amplification kit (Clontech, Japan). LvLRRFIP2-5′ RACE1 and LvLRRFIP2-3′ RACE1 primers (Table 1) were used for the first round 5′ end and 3′ end RACE-PCR using the following program: 94°C for 3 min, 10 cycles of 94°C for 30 s, 62°C for 30 s (a decrease of 0.5°C per cycle), 72°C for 2 min, 30 cycles of 94°C for 30 s, 57°C for 30 s, 72°C for 2 min, and a final extension at 72°C for 10 min. These PCR conditions were also applied to the second round 5′ end and 3′ end RACE PCR, where LvLRRFIP2-5′ RACE2 and LvLRRFIP2-3′ RACE2 primers were used, respectively. The PCR products were cloned into the pMD-20 vector (Takara, Japan), and then sequenced. The new sequences obtained in this study were deposited in the NCBI GenBank (http://www.ncbi.nlm.nih.gov/genbank/).

**Table 1:** PCR primers used in this study.

Primers	Primer sequences (5′-3′)
**For cDNA cloning**	
LvLRRFIP2-5′ RACE1	TCGTAGTCTCAGTTCTCTCGCTTCT
LvLRRFIP2-5′ RACE2	CGGGCCTCAGCCTCTTTT
LvLRRFIP2-3′ RACE1	GAAGGAACTAGAAGACCAAGTTAGAA
LvLRRFIP2-3′ RACE2	GCTCAGGATTCATTAGATGTTAGGTTA
**For Protein expression**	
pAcLvLRRFIP2-F1^a^	CGG**GGTACC**ATGCCTATAGTTAGAAAAGTCCCAG
pAcLvLRRFIP2-F2^a^	CGG**GGTACC**ATGAGGGCCGAATTGAAAG
pAcLvLRRFIP2-F3^a^	CGG**GGTACC**ATGGATGAGACGTCTAGGATGGA
pAcLvLRRFIP2-R1^a^	CGG**GGGCCC**TATCGAGCCGAGAGAGCATC
pAcLvLRRFIP2-R2^a^	CGG**GGGCCC**GAATCGGCCAATTCTGTCG
pAcLvLRRFIP2-R3^a^	CGG**GGGCCC**GGCAGGGTCTTGGGACAGGTCCT
**For qPCR**	
LvLRRFIP2-F	ACAGTTAGATAATGAAAAAGCGAC
LvLRRFIP2-R	GTGTTTCTTCAAGCTCGGTATACT
LvPEN2-F	GCATCAAGTTCGGAAGCTGT
LvPEN2-R	ACCCACATCCTTTCCACAAG
LvPEN4-F	ATGCTACGGAATTCCCTCCT
LvPEN4-R	ATCCTTGCAACGCATAGACC
LvALF1-F	ATAGTCGGGTTGTGGCACTC
LvALF1-R	GTCGTCCTCCGTGATGAGAT
LvCrustin-F	GGAGTAGGTGTTGGTGGTGGTT
LvCrustin-R	GCAGTCGCTTGTGCCAGTTC
LvLyz1-F	ACTGGTGCGGAAGCGACTA
LvLyz1-R	GGCGGATAGTCTCGGCG
LvLyz2-F	TCCCCCTGGTAAGAGAATCAAG
LvLyz2-R	GCACTTGGGCACCTGAGC
LvEF1α-F	GAAGTAGCCGCCCTGGTTG
LvEF1α-R	CGGTTAGCCTTGGGGTTGAG
**For RNAi**	
LvLRRFIP2i-F	GAAGGATAAGTATACCGAGCTTGAA
LvLRRFIP2i-R	GCCATATTCCTGTATTAACGCATCT
LvLRRFIP2i-T7F^b^	GGATCCTAATACGACTCACTATAGG GAAGGATAAGTATACCGAGCTTGAA
LvLRRFIP2i-T7R^b^	GGATCCTAATACGACTCACTATAGG GCCATATTCCTGTATTAACGCATCT
EGFP-F	GTTCAGCGTGTCCGGCGAG
EGFP-R	GTTCTTCTGCTTGTCGGCC
EGFP-T7F^b^	GGATCCTAATACGACTCACTATAGG TCAGCGTGTCCGGCGAG
EGFP-T7R^b^	GGATCCTAATACGACTCACTATAGG TCTTCTGCTTGTCGGCC

a Nucleotides in bold indicate restriction sites introduced for cloning.

b T7 RNA polymerase promoter sequence are underlined.

### Bioinformatics Analysis

The BLAST program (http://www.ncbi.nlm.nih.gov/BLAST/) was used to analyze the nucleotide sequence and to search for protein sequences from other species in the database. Multiple sequence alignments were performed using the ClustalX 2.0 program (http://www.ebi.ac.uk/tools/clustalw2). The simple modular architecture research tool (SMART, http://smart.embl-heidelberg.de) was used to analyze the protein domain topology. The neighbor-joining phylogenic trees were constructed based on the amino acid sequences using the MEGA 4.0 software (http://www.megasoftware.net/index.html) and bootstrapped for 1000 times.

### Immune Challenge and Gene Expression Analysis

Twelve kinds of tissues, namely, hemocytes, hepatopancreas, gill, heart, stomach, pyloric cecum, nerve, epithelium, eyestalk, intestine, seminal vesicle, and muscle, were obtained from healthy *L. vannamei* for RNA extraction. The RNeasy mini kit (Qiagen, Germany) was used to extract the total RNA from each tissue. The PrimeScript™ RT reagent kit (TaKaRa, Japan) was used to reverse transcribe the total RNA into first-strand cDNA for real-time quantitative PCR (qPCR) analysis. Primers LvLRRFIP2-F and LvLRRFIP2-R (Table 1) were used to detect the relative mRNA expression of LvLRRFIP2s in different tissues. LvLRRFIP2 expression was measured using the Master SYBR Green I system with the following program: one cycle at 95°C for 30 s, 40 cycles of 95°C for 5 s, 57°C for 30 s, and 78°C for 5 s. Three replicate qPCR analyses were performed per sample using Elongation factor 1α (EF1α) as internal control.

For the challenge experiments, healthy *L. vannamei* was intramuscularly injected with LPS (Sigma, USA) (2 µg/g), poly I:C (Sigma, USA) (2 µg/g), CpG-ODN2006 (Sigma, USA) (2 µg/g), *V. parahaemolyticus* (5.5×10^6^ CFU/g), *Staphylococcus aureus* (2.5×10^6^ CFU/g), and white spot syndrome virus (WSSV) (10^6^ copies/g) at the third abdominal segment. The *L. vannamei* injected with PBS were used as controls. Three animals from each group were randomly sampled for hemocyte collection at 0, 4, 8, 12, 24, 36, 48, and 72 h post-injection. The relative mRNA expression of the LvLRRFIP2 genes was detected by qPCR using the same program previously described.

### Plasmid Construction

The pAc5.1/V5-His A (Invitrogen, USA) and PCR products (amplified with primers pAcLvLRRFIP2-F1/pAcLvLRRFIP2-R1, pAcLvLRRFIP2-F2/pAcLvLRRFIP2-R2, and pAcLvLRRFIP2-F3/pAcLvLRRFIP2-R3, respectively) were digested with restriction enzymes Kpn I and Xba I (Takara, Japan) and purified to determine protein expression in S2 cells. The mixture was ligated at 4°C overnight, and then transformed into the DH5α competent cells. Positive clones were confirmed by colony PCR and sequenced. Green fluorescent protein (GFP) PCR products were inserted into pAc5.1/V5-His A at the Xba I and Sac II sites to construct pAc5.1-N-GFP for protein localization. LvLRRFIP2 DNA fragments were then inserted into pAc5.1-N-GFP at the Kpn I and Xba I sites. Luciferase reporter vectors using the promoter sequences of *Drosophila* antimicrobial peptides (AMPs), Attacin A (AttA), and Drosomycin (Drs), as well as *Penaeus monodon* AMP Penaeidin (PEN453 and PEN536) were constructed in our previous studies [19]–[24].

### Subcellular Localization Analysis of LvLRRFIP2

Given that no permanent shrimp cell line was available, *Drosophila* Schneider 2 (S2; Invitrogen, USA) cells were used for the functional and localization analysis of LvLRRFIP2s [26], [27]. S2 cells were seeded onto the cover slips in 12-well plates (TPP, Switzerland) for DNA transfection at 28°C in a *Drosophila* serum-free medium (SDM; Invitrogen, USA) supplemented with 10% fetal bovine serum (Invitrogen, USA) to perform localization analyses of LvLRRFIP2. The cells were then transfected with pAc5.1-N-GFP and pAc5.1-LvLRRFIP2-GFP using the Cellfectin II reagent (Invitrogen, USA) after 24 h. At 48 h post-transfection, cells on the cover slips were washed three times with PBS, fixed by Immunol Staining Fix Solution (Beyotime, China), and stained with Hoechst 33258 Solution (Beyotime, China). The treated cells were observed using a Leica laser scanning confocal microscope.

### Dual-luciferase Reporter Assays

For the dual-luciferase reporter assays, S2 cells were seeded overnight in 96-well plates (TPP, Switzerland) and transfected using 0.3 µg pAc5.1-LvLRRFIP2s, 0.2 µg reporter gene plasmids, and 0.02 µg pRL-TK *Renilla* luciferase plasmid (Promega, USA) in a well. The pRL-TK *Renilla* luciferase plasmid was used alone as an internal control. At 48 h post-transfection, firefly and *Renilla* luciferase activities were measured using the dual-luciferase reporter assay system (Promega, USA). All assays were performed with three independent transfections.

### Knockdown of LvLRRFIP2 in vivo by Double-stranded RNA (dsRNA)-mediated RNA Interference

The DsRNA of LvLRRFIP2 and EGFP were generated in vitro using gene specific primers (Table 1) according to T7 RiboMAX Express RNAi System (Promega, USA). For gene knockdown experiments, the experimental group (mean body weight 4 g to 5 g/*L. vannamei*) was intramuscularly injected with LvLRRFIP2 dsRNA (1 µg/g *L. vannamei*), whereas the control groups were injected with EGFP dsRNA and PBS only. Hemocyte samples from four animals of each treatment were collected at 0, 0.5, 1, 2, 3, 5, and 7 d post-dsRNA injections to determine the earliest time of maximal silencing. The total RNA was extracted and reverse transcribed to cDNA as previously described. The gene knockdown efficiency was checked using qPCR, and the optimum time of gene silencing was found. *L. vannamei* were challenged at the optimum time post-dsRNA injection.

### Bioassay of *V. parahaemolyticus*, *S. aureus*, WSSV, and PBS Challenge Tests in LvLRRFIP2 Knockdown *L. vannamei*

A total of 600 *L. vannamei* (mean body weight of 4 g to 5 g) were divided into four groups (150 specimens per group) for the *V. parahaemolyticus* (5.5×10^6^ CFU/g), *S. aureus* (2.5×10^6^ CFU/g), WSSV (10^6^ copies/g), and PBS challenges. Each group was further subdivided into three subgroups (50 specimens per group) for different dsRNA silencing treatments, i.e., injection with LvLRRFIP2 dsRNA (designated as dsLvLRRFIP2), EGFP dsRNA (designated as dsEGFP), or PBS. *L. vannamei* was then challenged with *V. parahaemolyticus*, *S. aureus*, WSSV, or PBS 2 d after dsRNA injection. The cumulative mortality was recorded every 8 h.

### Detection of AMP Gene Expression in LvLRRFIP2 Knockdown *L. vannamei*

Specific primers (Table 1) of AMP genes were designed based on published *L. vannamei* cDNA sequences of penaeidin2 (LvPEN2, GenBank No.AF390146), penaeidin4 (LvPEN4, GenBank No. AF390147), anti-lipopolysaccharide factor 1 (LvALF1, GenBank No.EW713395), crustin (LvCrustin, GenBank No.AY488496), lysozyme1 (LvLyz1, GenBank No.AY170126), and lysozyme2 (LvLyz2, GenBank No. JN039375). The expression level of these genes at detected times after PBS, dsEGFP, and dsLvLRRFIP2 challenge were measured using qPCR as previously described.

### Statistical Analysis

Student’s *t*-test was used to compare means from two samples using Microsoft Excel when applicable. In all cases, differences were considered significant at *p*<0.05. All experiments were repeated at least three times. The data were presented as the mean ± standard error (standard error of the mean, SEM). The Kaplan-Meier plot (log-rank *x* ^2^ test) was used to identify significant differences in mortality levels between the EGFP dsRNA and the LvLRRFIP2 dsRNA groups [28].

## Results

### cDNA Cloning and Bioinformatics Analysis of LvLRRFIP2

**Figure 1:**
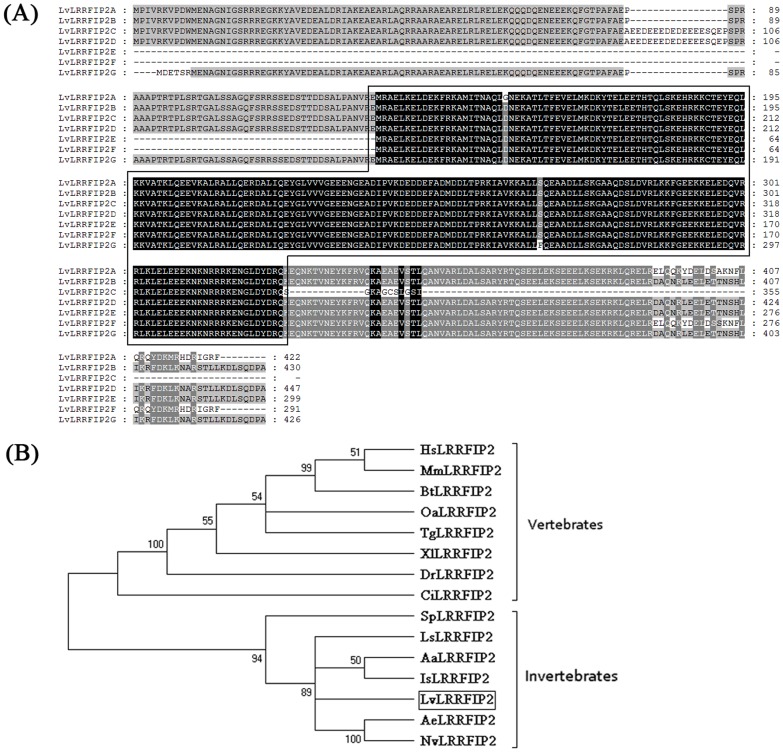
Multiple sequence alignment of LvLRRFIP2s in *Litopenaeus vannamei* and phylogenetic analysis of LRRFIP2 proteins from various species. (A) Multiple sequence alignment of LvLRRFIP2s in *Litopenaeus vannamei*. The identical amino acid residues shaded in black and the similar residues in gray. The DUF2051 domains are boxed. (B) Phylogenetic analysis of LRRFIP2 proteins. A rooted tree was constructed via the neighbor-joining method and was bootstrapped 1000 times using MEGA 4.0 (http://www.megasoftware.net/index.html). LvLRRFIP2 is boxed. LvLRRFIP2, *L. vannamei* LRRFIP2D (Accession No. JX840476); AeLRRFIP2, *Acromyrmex echinatior* LRRFIP2 (Accession No. EGI63253); NvLRRFIP2, *Nasonia vitripennis* LRRFIP2 (Accession No. XP_001608049); LsLRRFIP2; *Lepeophtheirus salmonis* LRRFIP2 (Accession No. ACO11882); AaLRRFIP2, *Aedes aegypti* LRRFIP2 (Accession No. XP_001654827); SpLRRFIP2, *Strongylocentrotus purpuratus* LRRFIP2 (Accession No. XP_782587); IsLRRFIP2, *Ixodes scapularis* LRRFIP2 (Accession No. XP_002410685); CiLRRFIP2, *Ciona intestinalis* LRRFIP2 (Accession No. XP_002130672); HsLRRFIP2, *Homo sapiens* LRRFIP2 (Accession No.NP_060194); BtLRRFIP2, *Bos taurus* LRRFIP2 (Accession No. NP_001033159); MmLRRFIP2, *Mus musculus* LRRFIP2 (Accession No. NP_082018); TgLRRFIP2, *Taeniopygia guttata* LRRFIP2 (Accession No. XP_002198991); DrLRRFIP2, *Danio rerio* LRRFIP2 (Accession No.NP_955773); XlLRRFIP2, *Xenopus laevis* LRRFIP2 (Accession No. NP_001085821); OaLRRFIP2, *Ornithorhynchus anatinus* LRRFIP2 (Accession No. XP_003430994).

Seven LvLRRFIP2 variants, namely, LvLRRFIP2A, LvLRRFIP2B, LvLRRFIP2C, LvLRRFIP2D, LvLRRFIP2E, LvLRRFIP2F, and LvLRRFIP2G, were found. The sequences at 5′ end of LvLRRFIP2G were distinct from that of the other six LvLRRFIP2 variants. The 5′ end sequences of LvLRRFIP2A, LvLRRFIP2B, LvLRRFIP2C, and LvLRRFIP2D were identical. Both LvLRRFIP2E and LvLRRFIP2F have the same 5′ end sequences, which were different from that of LvLRRFIP2A-D. The sequence details of these seven variants of LvLRRFIP2 are shown in Fig. S1 and Table 2. Multiple sequence alignment shows that LvLRRFIP2s are highly conserved with each other (Fig. 1A). The amino acid sequence was analyzed using the SMART program to determine the structural domains of LvLRRFIP2. All LvLRRFIP2s have a DUF2051 domain (Fig. 1A), which was found in a dsRNA binding protein named DUF2051, a novel protein that interacts with the LRR domain of human FliI protein [29]. The identities among LvLRRFIP2s ranged from 44% to 97% (Table 3). Compared with the LRRFIP2 proteins from other species, LvLRRFIP2 shares a 35% to 51% identity with the LRRFIP2 proteins from insect to human (Table 4). A phylogenetic tree was constructed to determine the evolutionary relationship of LvLRRFIP2 with other known LRRFIP2 molecules. The phylogenetic tree showed that LvLRRFIP2 belonged to the invertebrate group and was closely related to LRRFIP2 in *L. salmonis*, *Aedes aegypti*, *Acromyrmex echinatior*, *Ixodes scapularis*, and *Nasonia vitripennis*, which are all arthropods (Fig. 1B).

**Table 2:** The cDNA information of seven LvLRRFIP2 variants.

Name	GenBank accession No.	Full lengths	ORF lengths	Lengths of 5′ untranslated region	Lengths of 3′ untranslated region
LvLRRFIP2A	JX840473	1671 bp	1269 bp	50 bp	352 bp
LvLRRFIP2B	JX840474	1573 bp	1293 bp	50 bp	230 bp
LvLRRFIP2C	JX840475	1544 bp	1068 bp	50 bp	426 bp
LvLRRFIP2D	JX840476	1624 bp	1344 bp	50 bp	230 bp
LvLRRFIP2E	JX840477	1546 bp	900 bp	416 bp	230 bp
LvLRRFIP2F	JX840478	1639 bp	876 bp	416 bp	347 bp
LvLRRFIP2G	JX840479	1634 bp	1281 bp	123 bp	230 bp

**Table 3:** Identities of seven variants of LvLRRFIP2.

Seq	LvLRRFIP2A	LvLRRFIP2B	LvLRRFIP2C	LvLRRFIP2D	LvLRRFIP2E	LvLRRFIP2F	LvLRRFIP2G
LvLRRFIP2A	100%						
LvLRRFIP2B	95%	100%					
LvLRRFIP2C	75%	74%	100%				
LvLRRFIP2D	91%	95%	78%	100%			
LvLRRFIP2E	64%	69%	44%	66%	100%		
LvLRRFIP2F	68%	65%	45%	62%	93%	100%	
LvLRRFIP2G	92%	97%	71%	93%	69%	65%	100%

**Table 4:** Full-length amino acid sequence identities of LRRFIP2 in *L. vannamei* with other species.

Species	Accession number	Amina acids	Identity%
*Homo sapiens*	NP_060194	400	41%
*Mus musculus*	NP_082018	400	41%
*Bos taurus*	NP_001033159	400	41%
*Ornithorhynchus anatinus*	XP_003430994	280	40%
*Xenopus laevis*	NP_001085821	405	40%
*Taeniopygia guttata*	XP_002198991	399	41%
*Danio rerio*	NP_955773	405	40%
*Ciona intestinalis*	XP_002130672	429	35%
*Strongylocentrotus purpuratus*	XP_782587	459	39%
*Ixodes scapularis*	XP_002410685	386	41%
*Aedes aegypti*	XP_001654827	389	50%
*Lepeophtheirus salmonis*	ACO11882	406	39%
*Nasonia vitripennis*	XP_001608049	401	49%
*Acromyrmex echinatior*	EGI63253	402	51%

### Expression of LvLRRFIP2 in Healthy and Immune-challenged *L. vannamei*

**Figure 2:**
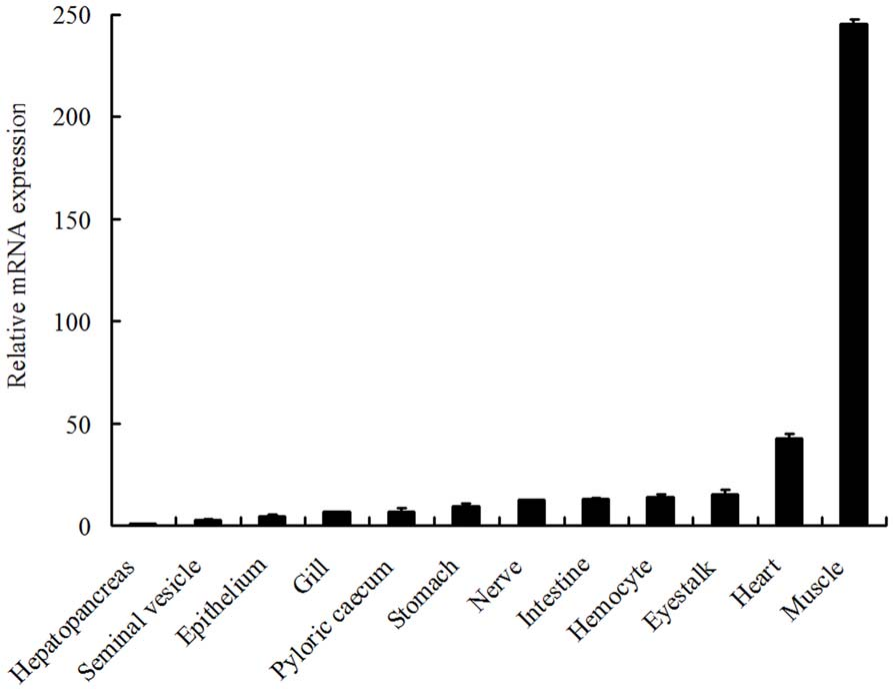
Tissue distributions of LvLRRFIP2s in healthy *L. vannamei*. Ten animals were used for tissue sampling. LvEF1α was used as the internal control to normalize the cDNA template used for real-time PCR analysis.

The primers LvLRRFIP2-F/LvLRRFIP2-R were designed according to the identical sequences among LvLRRFIP2s and were used to detect their total amount. The expression level of LvLRRFIP2s was highest in the muscle and lowest in hepatopancreas (Fig. 2). The ligands for TLR3 (poly I:C), TLR4 (LPS), TLR9 (CpG-ODN2006), gram-negative bacteria *V. parahaemolyticus*, gram-positive bacteria *S. aureus*, and one of the most common and most destructive viral pathogens in shrimp aquaculture, WSSV, were used for the challenge experiments [30], [31]. LvLRRFIP2s was highly expressed in hemocytes, which are important in immune response in *L. vannamei*. Thus, we selected hemocytes to study LvLRRFIP2s expression in response to immune challenges.

**Figure 3:**
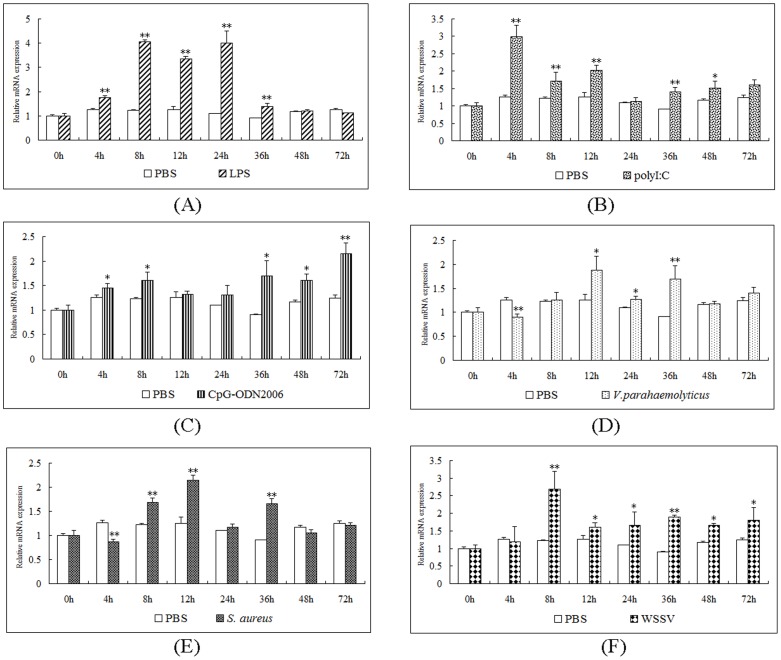
Temporal expression of LvLRRFIP2 in immune-challenged *L. vannamei*. The relative expression of LvLRRFIP2s in th treated groups ((A) lipope lysaccharide (LPS), (B) poly I:C, (C) CpG-ODN2006, (D) *Vibrio parahaemolyticus*, (E) *Staphyloccocus aureus*, (F) white spot syndrome virus (WSSV)) were compared with the control group. The relative expression level of the target genes was normalized to LvEF1α. The results were based on three independent experiments and expressed as mean values ± SD. Statistical significance was calculated using Student’s *t*-test (*indicates *p*<0.05 and **indicates *p*<0.01 compared with the control).

After LPS challenge, the level of LvLRRFIP2s increased to its peak at 8 h post-injection. After 48 h, the expression of LvLRRFIP2s was not obviously different from the control (Fig. 3A). After challenge with poly I:C, the LvLRRFIP2s expression was upregulated at all the detected times, with the highest expression level at 4 h post-injection (Fig. 3B). The LvLRRFIP2s expression was also upregulated by CpG-ODN2006 challenge (Fig. 3C). LvLRRFIP2s expression has short-term downregulation at 4 h post-injection, but upregulated after *V. parahaemolyticus* and *S. aureus* challenge (Fig. 3D, Fig. 3E). Compared with the control group, the WSSV-infected group showed increased LvLRRFIP2s expression starting at 8 h (Fig. 3F).

### Intracellular Localization of LvLRRFIP2

**Figure 4:**
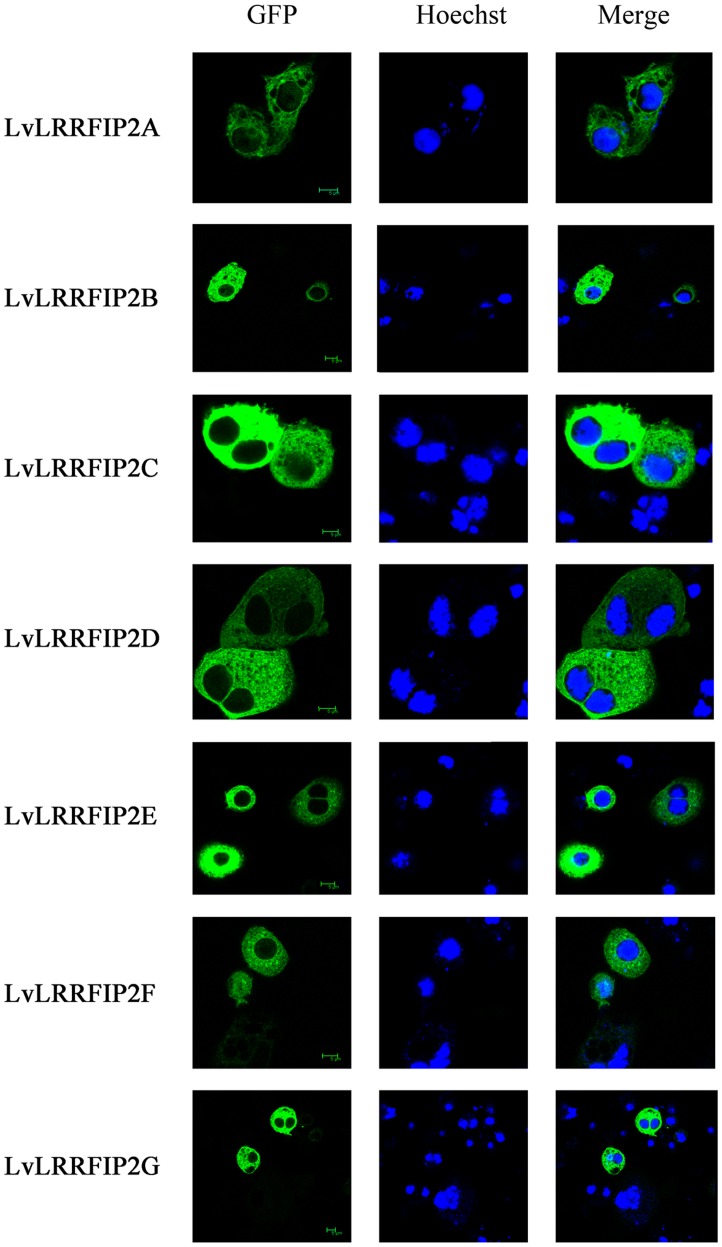
Subcellular localizations of LvLRRFIP2s. *Drosophila* S2 cells were transfected with GFP fusion proteins of pAcLvLRRFIP2s. At 48 h post-transfection, the cells were observed using a Leica laser scanning confocal microscope.

The subcellular location of LRRFIP2 has not been previously characterized. LvLRRFIP2s-GFP were observed under confocal microscopy using *Drosophila* S2 cells to identify the cellular localization of LvLRRFIP2. Although differences exist among the sequences of LvLRRFIP2s, their GFP fusion proteins were all observed in the cytoplasm of S2 cells (Fig. 4), which was consistent with the interaction of LRRFIP2 and MyD88, an adaptor protein downstream of TLRs [8].

### *Drosophila* and Shrimp AMP Genes were Regulated by LvLRRFIP2

**Figure 5:**
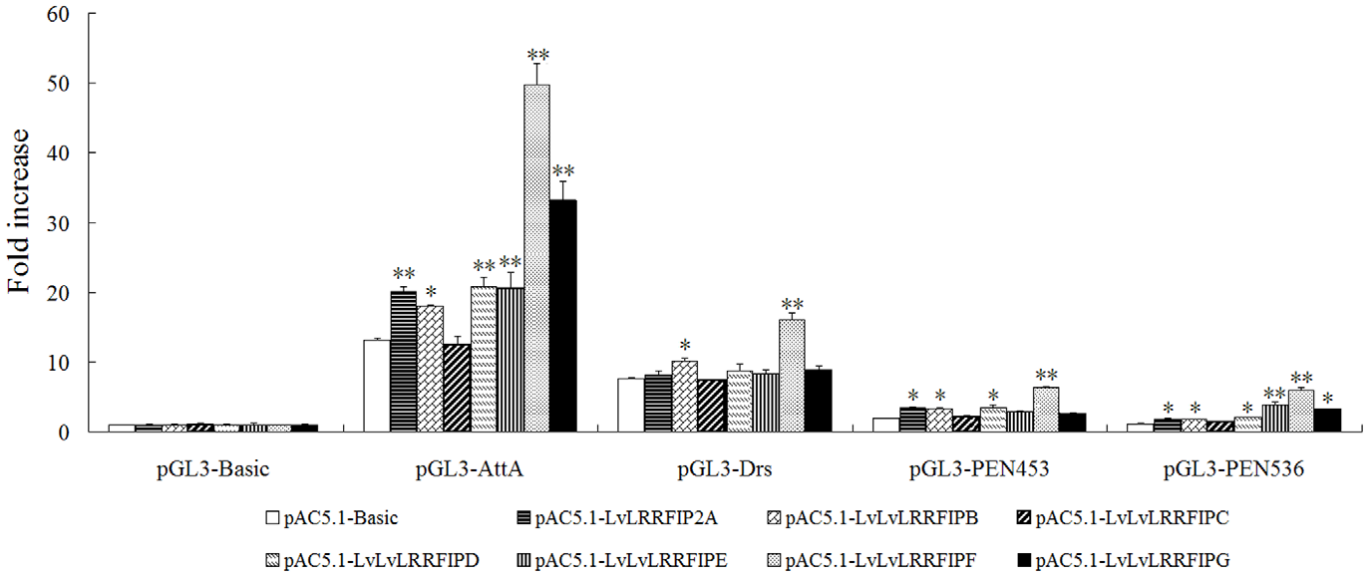
Effects of LvLRRFIP2s on the promoter activities of *Drosophila* and shrimp AMPs in *Drosophila* S2 cells. *Drosophila* S2 cells were transfected with the protein expression vector (pAC5.1 empty vector, one of the LvLRRFIP2s), the reporter gene plasmid (pGL3-Basic, pGL3-AttA, pGL3-Drs, pGL3-PEN453, or pGL3-PEN536), and the pRL-TK *Renilla* luciferase plasmid (as an internal control: Promega, USA). After 48 h, the cells were harvested for luciferase activity determination using the dual-luciferase reporter assay system (Promega, USA). All data are representative of three independent experiments. The bars indicate the mean ± SD of luciferase activity (*n* = 3). The statistical significance was calculated using Student’s *t*-test (*indicates *p*<0.05 and **indicates *p*<0.01compared with control).

LRRFIP2 is a positive regulator of NF-κB activity in murine macrophage cells [8]. AMPs are important immune factors in *Drosophila* and shrimp, and their expression is believed to be controlled mainly by the NF-κB signal pathway [20], [21], [32]. The NF-κB signal pathway can be activated by Toll, Pelle, TRAF6, Dorsal, and Relish in shrimp [19]–[24]. The present study demonstrated that LvLRRFIP2s activated the promoters of *Drosophila* and shrimp AMP genes. Compared with six other variants of LvLRRFIP2, LvLRRFIP2F induced higher activities of AMP promoters, including the *Drosophila* AMPs AttA (3.78-fold), Drs (2.11-fold), *L. vannamei* AMP PEN4 (3.32-fold), and *P. monodon* AMP PEN536 (5.14-fold) (Fig. 5).

### LvLRRFIP2 Suppression Led to an Increased Mortality of *L. vannamei* after *V. parahaemolyticus* Infection, but not after *S. aureus* and WSSV Infection

**Figure 6:**
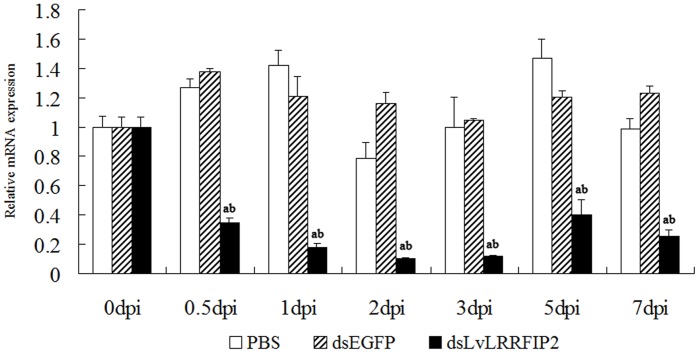
Expression of LvLRRFIP2 mRNA after knockdown by dsRNA-mediated RNAi. Injections of the enhanced EGFP dsRNA and PBS were used as dsRNA controls. Relative expression values were normalized to LvEF1α. The results are based on three independent experiments and expressed as mean values±SD. Statistical significance was calculated by the Student’s *t*-test (Letters a and b indicate *p*<0.05 compared with blank (0 h without any treatment) or PBS group, respectively).

DsRNA were used to knockdown all the variants of LvLRRFIP2. The relative expression level of LvLRRFIP2 in hemocytes after dsLvLRRFIP2 interference is shown in Fig. 6. Reduced LvLRRFIP2 mRNA expression was observed at 0.5 d post-injection. The most significant effect was detected 2 d post-injection. The relative expression of LvLRRFIP2 in dsLvLRRFIP2 injected group accounts for 10% of that of blank *L. vannamei* (0 h without any treatment) and 8% that of the PBS group. Compared with the dsLvLRRFIP2 injection group, the expression of LvLRRFIP2 was not significantly affected by dsEGFP and PBS injection at all the detected times (*p*>0.05).

**Figure 7:**
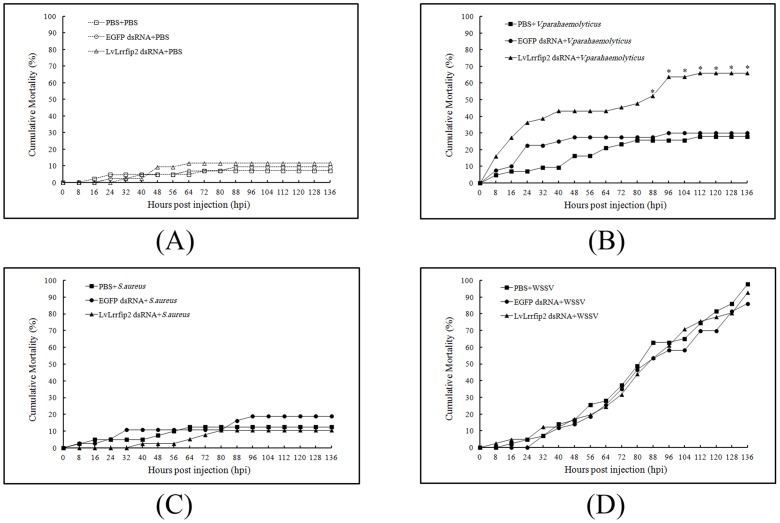
Gene silencing of *L. vannamei* LvLRRFIP2 increased its mortality after *V. parahaemolyticus* injection. *L. vannamei* were injected intramuscularly with PBS or dsRNAs corresponding to LvLRRFIP2 or EGFP. At 2 d after the initial injection, the animals were infected with PBS (negative control) (A), *V. parahaemolyticus* (B), *S. aureus* (C), or WSSV (D). Mortality was measured in each treatment group (n = 50) and was recorded every 8 h post-challenge. Differences in cumulative mortality levels between the LvLRRFIP2 dsRNA group and the EGFP dsRNA group were analyzed by Kaplan-Meier log-rank *χ* ^2^ tests. Significant differences in *L. vannamei* mortality are marked with asterisks, and were found only in *L. vannamei* challenged with *V. parahaemolyticus* from 88 hpi to the end of the experiment (*p*<0.05).

*L. vannamei* was challenged with *V. parahaemolyticus*, *S. aureus*, WSSV, and a PBS control to explore the possible involvement of LvLRRFIP2 in a protective response against invaders at 2 d post-dsRNA injection. The baseline cumulative mortality of *L. vannamei* injected with PBS at 2 d after LvLRRFIP2 dsRNA injection is shown in Fig. 7A. The final mortality rates at 136 hpi were low for all groups (11.6%, 9.3%, and 6.9% for the LvLRRFIP2 dsRNA, EGFP dsRNA, and PBS groups, respectively), and no significant difference in mortality was observed among the three groups (*p*>0.05). In the *V. parahaemolyticus* challenge test (Fig. 7B), the cumulative mortality of the LvLRRFIP2 dsRNA group began to increase at 8 h post-*V. parahaemolyticus* challenge. The cumulative mortality in the LvLRRFIP2 dsRNA group was significantly higher than in the EGFP dsRNA, starting at 88 hpi (Kaplan-Meier log-rank *χ* ^2^: 7.402, *p*<0.05). The final mortality rates at 136 hpi were 63.6%, 30.0%, and 27.9% for the LvLRRFIP2 dsRNA, EGFP dsRNA, and PBS groups, respectively. In the challenge tests with *S. aureus* and WSSV, no evidence showed that knockdown of LvLRRFIP2 expression by dsRNA had any statistically significance on cumulative mortality (Figs. 7C and 7D).

### The Expression of *L. vannamei* AMPs was Reduced by dsLvLRRFIP2 Interference

**Figure 8:**
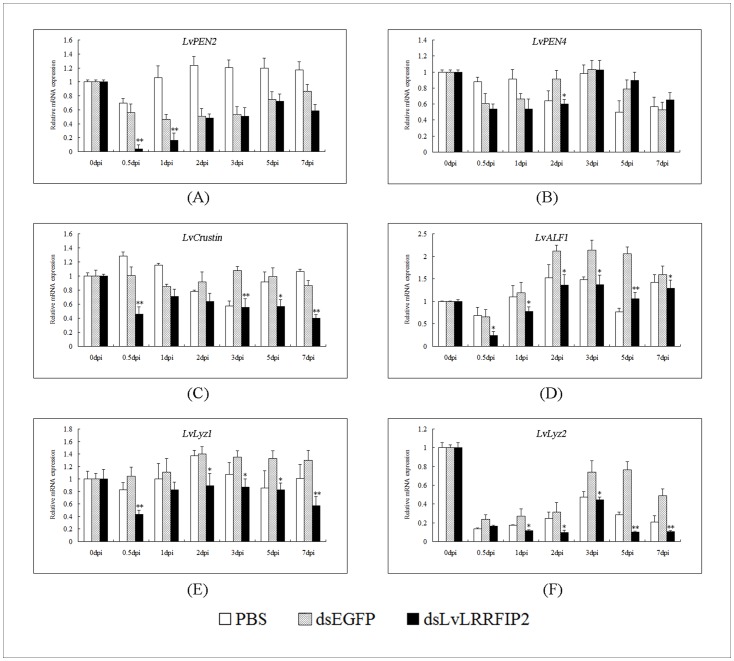
The expression of *L. vannamei* AMPs after dsLvLRRFIP2 was knocked down. The relative expression of the *L. vannamei* AMP genes ((A) LvPEN2, (B) LvPEN4, (C) LvCrustin, (D) LvALF1, (E) LvLyz1, (F) LvLyz2) were compared against the PBS and dsEGFP injection group at the corresponding times. Relative expression values were normalized to LvEF1α. The results are based on three independent experiments and expressed as mean values±SD. Statistical significance was calculated by the Student’s *t*-test (*indicates *p*<0.05 and **indicates *p*<0.01 compared with EGFP dsRNA injection group.

Considering that the knockdown of LvLRRFIP2 led to significantly increased mortality after *V. parahaemolyticus* infection (Fig. 7B), the expressions of six AMP genes were observed in LvLRRFIP2 knockdown *L. vannamei*. Fig. 8 shows that LvPEN4 underwent a brief period of downregulation at 0.5 d, 1 d, and 2 d after dsLvLRRFIP2 injection. However, the expression level of LvPEN4 was not significantly different in the dsLvLRRFIP2 and dsEGFP injection groups (Fig. 8B). Compared with the dsEGFP injection group, the expression level of LvPEN2, LvCrustin, LvALF1, LvLyz1, and LvLyz2 decreased at all detected times in the dsLvLRRFIP2 injection group (Figs. 8A, 8C, 8D, 8E, and 8F, respectively). All the results corresponded to the increase of the cumulative mortality of *L. vannamei* in the LvLRRFIP2 dsRNA group challenged with *V. parahaemolyticus*.

## Discussion

LRRFIP2 is a poorly characterized protein implicated in TLR responses [8]. To date, no report about the function of LRRFIP2 protein in invertebrates exists. In the present study, seven variants of LRRFIP2 were cloned from *L. vannamei*. The LvLRRFIP2 expression was regulated by different immune challenges. Luciferase reporter assays showed that *Drosophila* and shrimp AMP genes can be regulated by LvLRRFIP2. The knockdown of LvLRRFIP2 increased the cumulative mortality of *L. vannamei* after *V. parahaemolyticus* infection, but not after *S. aureus* and WSSV infection. All these results suggest that LvLRRFIP2 has a function in antibacterial response.

The analysis of all LRRFIP2 proteins presently published on the NCBI database showed that several isoforms of LRRFIP2 exist in *Nasonia vitripennis*, *Danio rerio*, and 12 kinds of mammals, including human and murine. The differences among isoforms in one of these species can be divided into three groups: 1) the nucleotide sequences at the 5′ end were different; 2) the nucleotide sequences at the 3′ end were different; and 3) several nucleotide sequences were missing in the short isoforms. *N. vitripennis* was the only invertebrate variant of LRRFIP2 found. In *N. vitripennis*, nucleotide sequences of the two LRRFIP2 isoforms were different only at the 5′ end. In the present study, seven variants of LvLRRFIP2 were cloned in *L. vannamei*. Most of the nucleotide sequences were identical in the seven variants of LvLRRFIP2, except for the different nucleotide fragments at the 5′ end and the lacked sequences in the short isoforms. Unlike LRRFIP2 in vertebrate species with varied isoforms, different nucleotide sequences at the 3′ end were not found in LvLRRFIP2s in this study. The seven variants of LvLRRFIP2 were different isoforms of LRRFIP2 gene in *L. vannamei*.

qPCR analysis showed that LvLRRFIP2 was detected in all tissues examined in *L. vannamei*, indicating a ubiquitous and constitutive expression of LvLRRFIP2 (Fig. 2). LvLRRFIP2s was expressed significantly higher in the muscle than the other tissues, which is similar to the expression pattern of the human LRRFIP2 gene [9]. Considering that LRRFIP2 is a protein implicated in TLR responses [8], the expression of LvLRRFIP2 was investigated in the hemocytes of *L. vannamei* after stimulation with the ligands of different TLRs, gram-negative bacterium *V. parahaemolyticus*, gram-positive bacterium *S. aureus*, and viral pathogen WSSV to improve understanding of the functions of LRRFIP2. In humans, LRRFIP2 mRNA expression level is regulated by LPS stimulation [8]. LvLRRFIP2 was upregulated by LPS stimulation in *L. vannamei*, especially at 8 h to 24 h after LPS challenge. LvLRRFIP2 was also upregulated at different levels after challenged by poly I:C, CpG-ODN2006, *V. parahaemolyticus*, *S. aureus*, and WSSV. These data indicate that LvLRRFIP2 participates in the immune response of *L. vannamei*.

We analyzed the subcellular localization to better define LvLRRFIP2. LRRFIP1 is the related gene of LRRFIP2 and is predominantly dispersed in the cytoplasm of primary murine monocytes [11], [33]. Subcellular localization of the seven variants of LvLRRFIP2 proteins were all observed in the cytoplasm of the S2 cells. This observation is consistent with the putative function of LRRFIP2 as an interactional protein with MyD88 in the modulation of TLR signaling [8]. The present study is the first to report on the subcellular localization of the LRRFIP2 protein. However, the function of LRRFIP2 needs further investigation.

In *Drosophila*, the Toll pathway is involved in immune response regulation, which controls several immune-related genes, including AMP genes, whose expression was believed to be controlled mainly by the NF-κB signal pathway [19], [27], [32]. A similar regulation mechanism for the shrimp AMPs exists [21], [22]. As MyD88 interactors, both LRRFIP1 and LRRFIP2 are positive regulators of the NF-κB activities in human [8]. The present study reveals that LvLRRFIP2 can activate the promoters of *Drosophila* and shrimp AMP genes in *Drosophila* S2 cells, suggesting that LvLRRFIP2 displays antibacterial function by regulating the expression of AMPs through the Toll pathway. LvLRRFIP2 was knocked down and the cumulative mortality of *L. vannamei* upon *V. parahaemolyticus*, *S. aureus*, and WSSV infection were detected to further study the function LRRFIP2 in the immune pathway of *L. vannamei*. The cumulative mortality of *L. vannamei* significantly increased upon *V. parahaemolyticus* infection. The expression of *L. vannamei* AMPs was reduced by dsLvLRRFIP2 interference, which corresponded with the increased cumulative mortality of *L. vannamei* in the LvLRRFIP2 dsRNA group challenged by *V. parahaemolyticus*. Thus, we speculated that the knockdown of LvLRRFIP2 impaired the immune defense of invaded *V. parahaemolyticus* by reducing the AMP expression. The cumulative mortalities of *L. vannamei* were not significantly changed upon *S. aureus* and WSSV infection when LvLRRFIP2 was knocked down. However, we cannot analyze the cause according to the present results, thus future studies are needed. In summary, the results suggest that LvLRRFIP2 has a function in the innate immune pathway in *L. vannamei*, at least against *V. parahaemolyticus*.

## Supporting Information

Figure S1 **Nucleotide and deduced amino acid sequences of LvLRRFIP2s from** ***Litopenaeus vannamei*** **.** (A) LvLRRFIP2A, (B) LvLRRFIP2B, (C) LvLRRFIP2C, (D) LvLRRFIP2D, (E) LvLRRFIP2E, (F) LvLRRFIP2F, (G) LvLRRFIP2G. The nucleotide (lower row) and deduced amino acid (upper row) sequences are shown and numbered on the left. The initiation codon (ATG) and stop codon (TAA or TGA) are in boldface. The DUF2051 domains are shaded. Different sequences at the 5′ end were boxed and painted by different colors. The sequences missing in several LvLRRFIP2s were underlined.
(TIF)
