# Supplementary figures and images for: Identification and Function of Leucine-Rich Repeat Flightless-I-Interacting Protein 2 (LRRFIP2) in Litopenaeus vannamei
Source: PLoS One. 2013 Feb 28;8(2):e57456. doi: 10.1371/journal.pone.0057456 (PMC3585381; doi:10.1371/journal.pone.0057456)

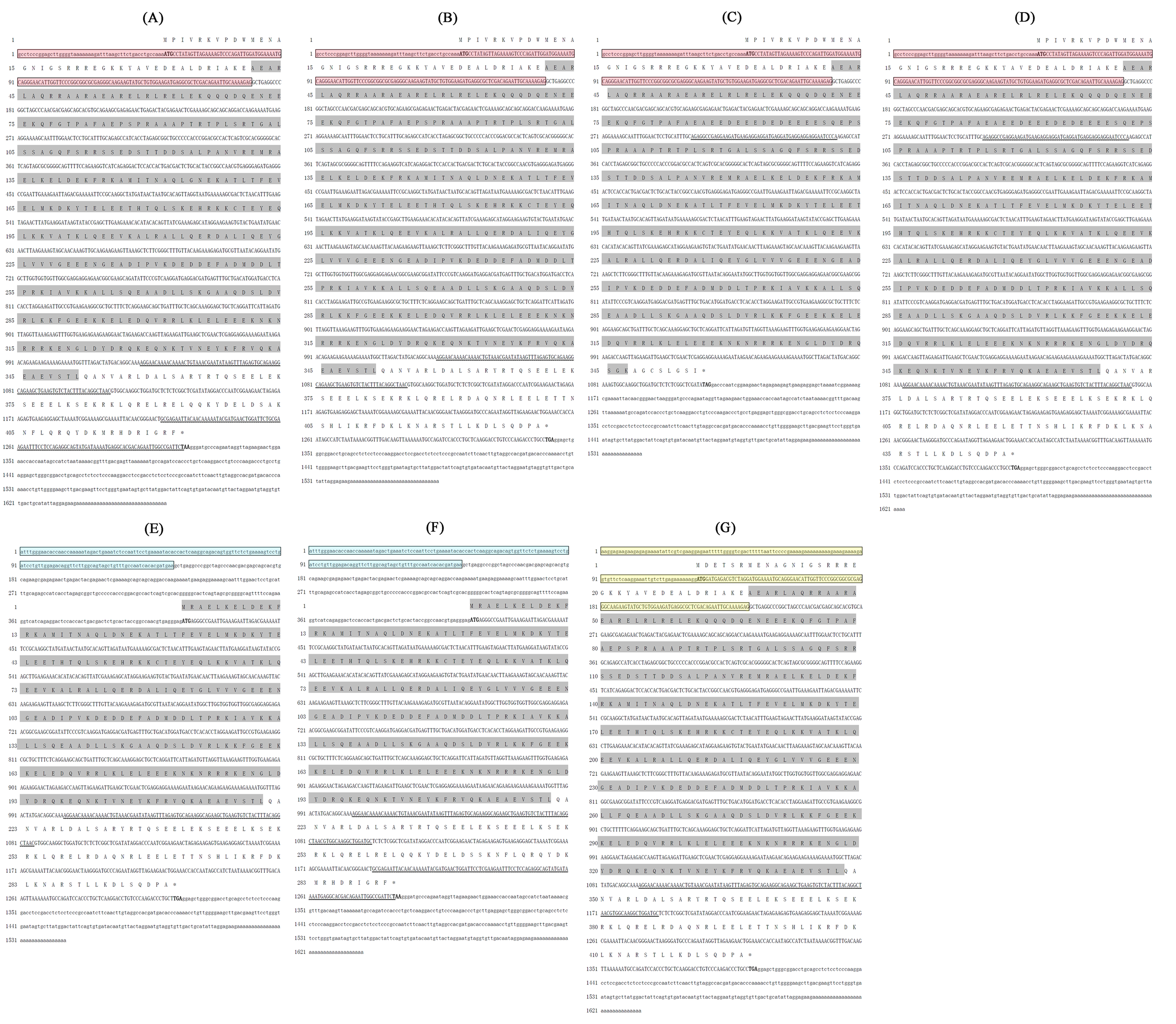

Supplement: Figure S1 — Nucleotide and deduced amino acid sequences of LvLRRFIP2s from Litopenaeus vannamei . (A) LvLRRFIP2A, (B) LvLRRFIP2B, (C) LvLRRFIP2C, (D) LvLRRFIP2D, (E) LvLRRFIP2E, (F) LvLRRFIP2F, (G) LvLRRFIP2G. The nucleotide (lower row) and deduced amino acid (upper row) sequences are shown and numbered on the left. The initiation codon (ATG) and stop codon (TAA or TGA) are in boldface. The DUF2051 domains are shaded. Different sequences at the 5′ end were boxed and painted by different colors. The sequences missing in several LvLRRFIP2s were underlined. (TIF) [file pone.0057456.s001.tif]
